# Malaria in the Time of COVID-19: Do Not Miss the Real Cause of Illness

**DOI:** 10.3390/tropicalmed6020040

**Published:** 2021-03-26

**Authors:** Johannes Jochum, Benno Kreuels, Egbert Tannich, Samuel Huber, Julian Schulze zur Wiesch, Stefan Schmiedel, Michael Ramharter, Marylyn M. Addo

**Affiliations:** 1 Department of Tropical Medicine, Bernhard Nocht Institute for Tropical Medicine & I. Department of Medicine, University Medical Center Hamburg-Eppendorf, 20359 Hamburg, Germany; b.kreuels@uke.de (B.K.); ramharter@bnitm.de (M.R.); 2 National Reference Centre for Tropical Pathogens, Bernhard Nocht Institute for Tropical Medicine, 20359 Hamburg, Germany; tannich@bnitm.de; 3 I. Department of Medicine, University Medical Center Hamburg-Eppendorf, 20246 Hamburg, Germany; s.huber@uke.de (S.H.); j.schulze-zur-wiesch@uke.de (J.S.z.W.); s.schmiedel@uke.de (S.S.); m.addo@uke.de (M.M.A.)

**Keywords:** severe acute respiratory syndrome coronavirus 2, plasmodium falciparum, differential diagnosis, cognitive bias, diagnostic reasoning

## Abstract

We report a case of *Plasmodium falciparum* malaria in a patient asymptomatically co-infected with severe acute respiratory syndrome coronavirus 2 (SARS-CoV-2). In the current ongoing coronavirus pandemic, co-infections with unrelated life-threatening febrile conditions may pose a particular challenge to clinicians. The current situation increases the risk for cognitive biases in medical management.

Since the end of 2019, the global outbreak of coronavirus disease 2019 (COVID-19) has spread rapidly and represents a major challenge to many healthcare systems worldwide [1]. In this situation, co-infections and comorbidities pose a particular challenge to clinicians, as the current focus on COVID-19 management may, on the one hand, lead to inappropriate diagnostics and management of other medical conditions, and on the other hand, to the risk of propagation of severe acute respiratory syndrome coronavirus 2 (SARS-CoV-2) within hospitals. Here, we describe a case of acute falciparum malaria and concurrent SARS-CoV-2 infection, illustrating the challenges in diagnostic reasoning during the current pandemic.

A 61-year-old female patient presented at the emergency department of the University Medical Center Hamburg-Eppendorf on 22 March 2020 with a fever of 40.2 °C, myalgia, and diarrhea. All symptoms had been present for four days. Nine days before presentation, she had returned from a two-week journey to Cameroon. She reported no chronic medical conditions or regular medication. Due to her recent air travel, fever and the ongoing pandemic with COVID-19, she was tested for SARS-CoV-2. Reverse transcription PCR of an oropharyngeal swab turned out positive and the patient was admitted to the COVID-19 isolation ward. Auscultation, lung ultrasound, and chest X-ray revealed no findings consistent with an atypical pneumonia (Figure 1). Laboratory results showed severe thrombocytopenia of 23,000/µL and C-reactive protein of 103 mg/L (Table 1). These results were considered atypical for a clinically mild case of COVID-19, given the available data [2]. Therefore, differential diagnoses were sought.

Prior to admission, the patient herself had already initiated presumptive treatment for malaria with atovaquone-proguanil due to her recent travel and lack of chemoprophylaxis. As history and laboratory findings were, indeed, compatible with malaria, we performed a microscopic examination of the patient’s blood. A thin blood film was found to be positive for *Plasmodium falciparum* with an asexual parasitemia of 4% (Figure 2). Treatment with atovaquone-proguanil was continued and all clinical symptoms subsided on day three after admission. Two sets of blood cultures and PCR diagnostic for influenza remained negative. Although the detection of SARS-CoV-2 was confirmed in a subsequent sample, the patient never experienced respiratory symptoms during the follow-up of one month.

Due to its increasingly wide spread distribution in the population, COVID-19 may be encountered as a co-infection with any other classical disease. Dual infection with SARS-CoV-2 and malaria have been reported recently, with most patients being free of respiratory symptoms attributable to SARS-CoV-2 [3,4]. It is of importance not to miss such co-infections to avoid in-hospital spread of SARS-CoV-2 infection with the potential for high mortality in other hospitalized patients. At the same time, it is important not to narrow the spectrum of differential diagnoses of febrile conditions despite the ongoing COVID-19 pandemic. When patient load is high and staff are in short supply, it is tempting to be satisfied with an initial diagnosis, particularly when this diagnosis is consistent with the current outbreak. The pandemic situation contributes to the risk for cognitive biases during the medical management, in particular, availability bias and premature closure [5,6]. 

Importantly, only one day after admission of our patient, we received a telephone call by a desperate man who was denied a medical evaluation when he developed a high fever after travel to Ghana. His family doctor, as well as the local hospital, judged this to likely be COVID-19 and recommended home isolation—advice that has potentially fatal consequences. Subsequently, this patient was also admitted to hospital with *Plasmodium falciparum* malaria. The two patients illustrate the currently increased risk of missing life-threatening differential diagnoses with the requirement of a specific therapy beyond giving antibiotics for suspected sepsis. This becomes even more relevant when international travel is relaunched and is an important lesson for future outbreaks.

## Figures and Tables

**Figure 1:**
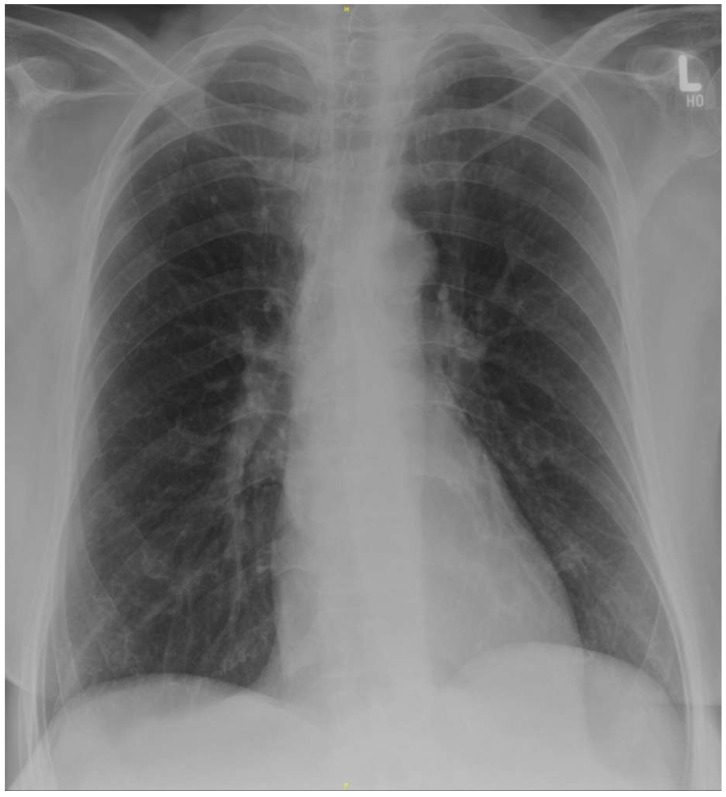
Supine chest X-ray at the time of admission without evidence of pneumonic consolidations or ground glass opacities. Courtesy of Prof. Gerhard Adam, Department of Diagnostic and Interventional Radiology and Nuclear Medicine, University Medical Center Hamburg-Eppendorf, Hamburg.

**Figure 2:**
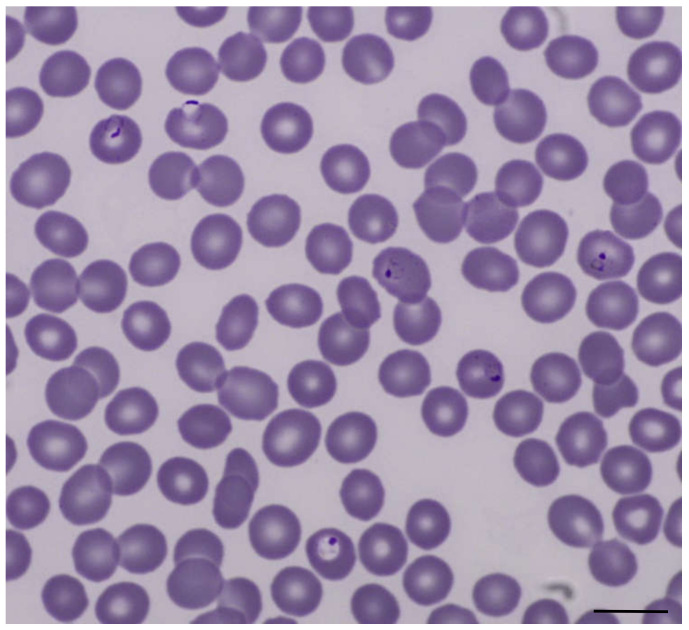
Thin blood film with *Plasmodium falciparum* trophozoites, *Giemsa* stain, original magnification 1000×. Scale bar: 10 µm

**Table 1:** Laboratory values of the patient on admission, 4 days after onset of symptoms.

Laboratory Parameter and Unit	Patient Value	Reference Range
Leucocyte count/µL	2700	3800–11,000
Hemoglobin g/dL	13.9	12.3–15.3
Platelet count/µL	23,000	150,000–400,000
Total bilirubin mg/dL	1.5	0.3–1.2
Alanine aminotransferase U/L	40	<35
Aspartate aminotransferase U/L	36	<35
Lactate dehydrogenase U/L	358	120–246
Creatinine mg/dL	0.67	0.55–1.02
C-reactive protein mg/L	103	<5

## Data Availability

Not applicable.
